# Supplementary material for: Palmitic Acid Inhibits Myogenic Activity and Expression of Myosin Heavy Chain MHC IIb in Muscle Cells through Phosphorylation-Dependent MyoD Inactivation
Source: Int J Mol Sci. 2023 Mar 19;24(6):5847. doi: 10.3390/ijms24065847 (PMC10054354; doi:10.3390/ijms24065847)
Supplement: Supplementary file 1 [file ijms-24-05847-s001.zip › Supplementary Table S1.pdf]

Supplementary Table S1. Sequences of primers used for real-time PCR

Gene and encoded protein names		
Myod	Forward	CCCCGGCGGCAGAATGGCTACG
(MyoD)	Reverse	GGTCTGGGTTCCCTGTTCTGTGT
Myog	Forward	ACTCCCTTACGTCCATCGTG
(Myogenin)	Reverse	CAGGACAGCCCCACTTAAAA
Myh7	Forward	CCAAGGGCCTGAATGAGGAG
(MHC I)	Reverse	GCAAAGGCTCCAGGTCTGAG
Myh2	Forward	AAGCGAAGAGTAAGGCTGTC
(MHC IIa)	Reverse	TGATTGCTTGCAAAGGAAC
Myh1	Forward	AGGCCAGGGTCCGTGAA
(MHC IIx)	Reverse	CCACGTTGCGCTTCTGTTC
Myh4	Forward	CCGAGCAAGAGCTACTGGA
(MHC IIb)	Reverse	TGTTGATGAGGCTGGTGTTC
Gapdh	Forward	GTCGTGGATCTGACGTGCC
(GAPDH)	Reverse	ATGCCTGCTTCACCACCTTC

MHC; myosin heavy chain, GAPDH; glyceraldehyde-3-phosphate dehydrogenase
