# Supplementary figures and images for: Palmitic Acid Inhibits Myogenic Activity and Expression of Myosin Heavy Chain MHC IIb in Muscle Cells through Phosphorylation-Dependent MyoD Inactivation
Source: Int J Mol Sci. 2023 Mar 19;24(6):5847. doi: 10.3390/ijms24065847 (PMC10054354; doi:10.3390/ijms24065847)

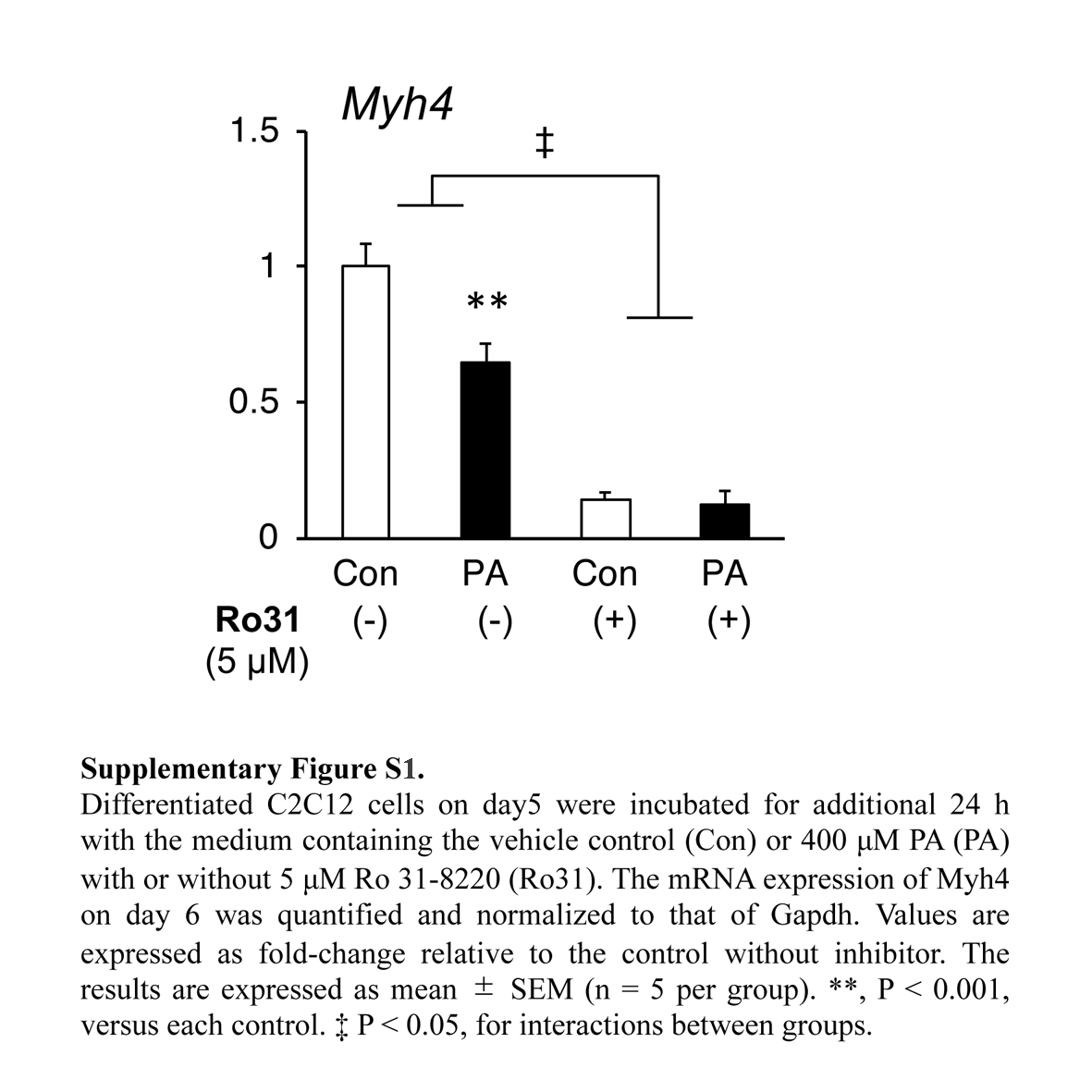

Supplement: Supplementary file 1 [file ijms-24-05847-s001.zip › Supplementary Figure S1.tif]

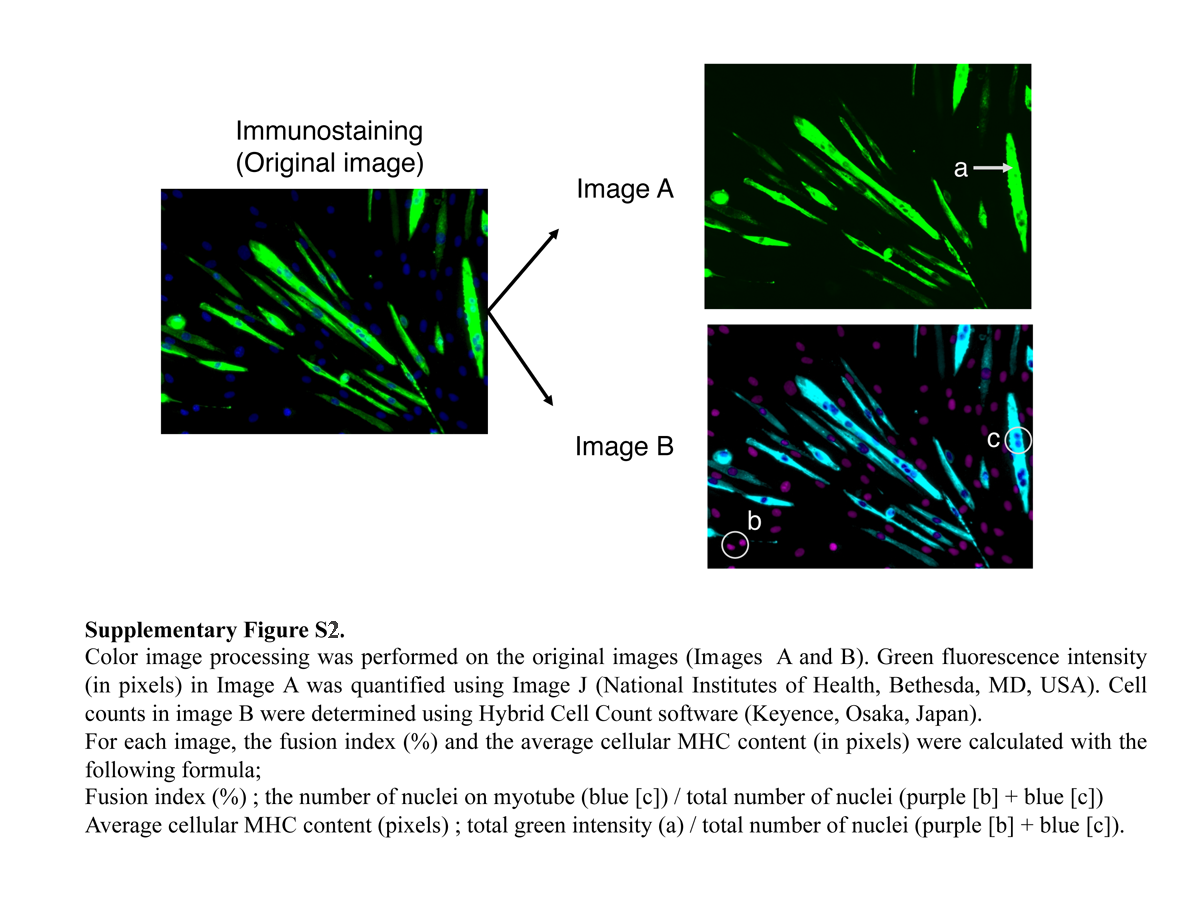

Supplement: Supplementary file 1 [file ijms-24-05847-s001.zip › Supplementary Figure S2.tif]
